# Hyperglycemia Is Not Associated With Higher Volumetric BMD in a Chinese Health Check-up Cohort

**DOI:** 10.3389/fendo.2021.794066

**Published:** 2022-01-03

**Authors:** Ling Wang, Kaiping Zhao, Xiaojuan Zha, Limei Ran, Heng Su, Yingying Yang, Qing Shuang, Yandong Liu, Li Xu, Glen M. Blake, Xiaoguang Cheng, Klaus Engelke, Annegreet Vlug

**Affiliations:** ^1^ Department of Radiology, Beijing Jishuitan Hospital, Beijing, China; ^2^ Department of Medical Record Management and Statistics, Beijing Jishuitan Hospital, Beijing, China; ^3^ Department of Health Management, Yijishan Hospital of Wannan Medical College, Wuhu, China; ^4^ Department of Health Management, Affiliated Hospital of Guizhou Medical University, Guiyang, China; ^5^ Department of Endocrinology, The First People’s Hospital of Yunnan Province, Kunming, China; ^6^ School of Biomedical Engineering and Imaging Sciences, King’s College London, St Thomas’ Hospital, London, United Kingdom; ^7^ Department of Medicine 3, FAU University Erlangen-Nürnberg and Universitätsklinikum Erlangen, Erlangen, Germany; ^8^ Institute of Medical Physics, University Erlangen-Nürnberg, Erlangen, Germany; ^9^ Center for Bone Quality, Department of Internal Medicine, Division of Endocrinology, Leiden University Medical Center (LUMC), Leiden, Netherlands

**Keywords:** fasting plasma glucose (FPG), type 2 diabetes, osteoporosis, areal bone mineral density (aBMD), volumetric bone mineral density (vBMD)

## Abstract

**Background and Purpose:**

Type 2 diabetes mellitus patients have an increased fracture risk despite having higher areal bone mineral density (aBMD) measured by DXA. This apparent paradox might be explained by the overestimation of BMD by DXA due to the higher fat mass in type 2 diabetes mellitus patients. Volumetric BMD (vBMD) as assessed by quantitative CT (QCT) is not influenced by fat mass. We assessed the association of vBMD and fasting plasma glucose in a large cohort of Chinese subjects and compared the vBMD in healthy and diabetic subjects. In addition, we compared the relation between aBMD, vBMD, glucose and fat mass in a subset of this cohort.

**Materials and Methods:**

10309 participants from the China Biobank project underwent QCT based on chest low dose CT to compute vBMD of L1 and L2 vertebrae and FPG measurements between 2018 and 2019. Among them, 1037 subjects also had spine DXA scans. Data was analyzed using linear regression models.

**Results:**

In the total cohort (5889 men and 4420 women, mean age 53 years, range 30-96), there was no significant association between vBMD and FPG after adjustment for age (women: p=0.774; men: p=0.149). 291 women and 606 men fitted the diagnostic criteria of diabetes. Both women and men with diabetes had lower vBMD compared to non-diabetic subjects, but this became non-significant after adjusting for age in the total cohort (women: p=0.817; men: p=0.288) and after propensity score matching based on age (women: p=0.678; men: p=0.135). In the DXA subcohort, aBMD was significantly higher in men with diabetes after adjusting for age and this difference disappeared after further adjusting for total fat area (p=0.064).

**Conclusion:**

We did not find any effect of fasting plasma glucose or diabetes on the volumetric BMD measured with QCT after adjustment for age. Therefore, vBMD measured with QCT might be a more reliable measurement to diagnose osteoporosis and assess fracture risk than aBMD measured with DXA in diabetic patients.

## 1 Introduction

Although type 2 diabetes mellitus and osteoporosis are common diseases in the ageing society, the relationship between these is less clear (1). Accumulating data has shown that the risk of osteoporotic fractures is increased in DM patients (2–5); a recent meta-analysis showed an increase in the risk of hip fracture in diabetes (type 1: relative risk (RR) 4.93, CI 3.06-7.95 and type 2: RR 1.33, CI 1.19-1.49) and for non-vertebral fractures (type 1: RR 1.92, CI 0.92-3.99 and type 2: RR 1.19, CI 1.11-1.28) (2). Contrary to the association between low bone mineral density (BMD) and diabetes consistently observed in type 1 DM patients, there is increasing evidence from recent studies indicating that type 2 diabetes mellitus patients have higher BMD compared to healthy subjects (1, 5, 6). Since higher BMD is associated with lower fracture risk in the general population, this apparent paradox might be explained by the overestimation of areal BMD (aBMD) by dual energy X-ray absorptiometry (DXA), the standard measurement method of BMD in clinical practice due to the higher fat mass in type 2 diabetes mellitus patients. Fracture risk prediction in type 2 diabetes mellitus becomes more challenging, since most fracture risk calculators, such as the FRAX tool, therefore underestimate fracture risk for individuals with diabetes due to this higher BMD (6). Furthermore, the associated under-treatment of bone fragility in type 2 diabetes mellitus patients could lead to inadequate fracture prevention (7).

DXA is a projectional method thus aBMD measurements are subject to variations in soft tissue thickness and composition. Algorithms used in commercial DXA scanners are based on assumptions about the homogenous disposition of fat in the body that are not generically valid (8). For example, obesity increases the likelihood of vertebral fracture but aBMD is known to increase with body weight in subjects with higher BMI. Volumetric BMD (vBMD in units of mg/cm^3^) measured by quantitative computed tomography (QCT) is a three-dimensional measure that is much less affected by body size and soft tissue composition. However, vBMD has been sparsely applied in the investigation of the relationship between BMD and type 2 diabetes mellitus because it is less frequently performed in the clinical investigation of osteoporosis. It is still unknown whether vBMD measured with QCT is a better indicator of true skeletal status than aBMD in patients with diabetes.

Therefore, in the present study we investigate the relation between vBMD measured with QCT and fasting plasma glucose in a large cohort of Chinese subjects and compare the vBMD between subjects with and without diabetes. In addition, we aim to directly compare the association of vBMD and aBMD in subjects with and without diabetes and we hypothesize that body fat influences the association of aBMD and diabetes more than vBMD.

## 2 Materials and Methods

### 2.1 Participants

Participants included in this study were a subset of the China Biobank project, a prospective nationwide multi-center cohort study studying osteoporosis, obesity, and fatty liver (6). This cohort has been registered with the US clinical trials database (clinicaltrials.gov; trial identifier: NCT03699228). Subjects in the present study were originally referred to the health management centers of the affiliated Yijishan Hospital of Wannan Medical University (4142 women, 5501men), and the affiliated hospital of Guiyang Medical University (278 women, 388 men), as part of their employers’ health check-up programs, and received a low dose chest CT (LDCT) scan for lung cancer screening. A total of 5889 men and 4420 women were included in the study, which involved the post-scan processing of CT (QCT full cohort). No additional radiation was involved. Among the study participants, 444 women and 593 men had DXA scans of the lumbar spine (DXA subcohort). The study was approved by the ethics committee of Beijing Jishuitan hospital and each participant gave written informed consent for their data to be used.

### 2.2 Blood Sampling and Laboratory Analysis

The blood sampling and laboratory analysis are part of the health checkup procedure and were described in detail previously (9). After an overnight fast, blood samples were drawn and fasting plasma glucose (FPG) concentration was measured using the hexokinase method. All tests and analyses were conducted in a certified clinical examination center at each of the collaborating medical centers. Diabetes was defined as FPG ≥ 7.0 mmol/L according to the diagnostic criteria of the American Diabetes Association (10) and/or use of antihyperglycemic medication and/or self-reported diagnosis of diabetes.

### 2.3 Anthropometry and Other Covariates

Weight (kg) and height (m) were measured using calibrated digital scales and stadiometers and body mass index was calculated [BMI = weight (kg)/height (m)^2^]. Information on antidiabetic medication was restricted to insulin and/or oral antidiabetic medications or no medication use. Total abdominal fat area (TFA) was determined at the level of the 2nd lumbar vertebra (L2) by CT.

### 2.4 QCT and DXA Scans

The details of the China Biobank study protocol have been published elsewhere (9). LDCT scans were conducted on an Optima CT540 CT scanner (GE Healthcare, WI, USA) at the Wannan center and a Supria CT scanner (Hitachi, Tokyo, Japan) at the Guiyang center. The LDCT was performed according to the same protocol at both centers. Mindways QCT Pro (Mindways Software, Inc., Austin, TX, USA) was used for all QCT vBMD measurements and all CT scans were acquired at 120 kVp. LDCT is now the standard for lung cancer screening and the subsequent analysis of these CT scans enabled evaluation of vBMD at L1 and L2 using the Mindways QCT Pro software calibrated with a QCT asynchronous phantom (Mindways, Austin, TX, USA). Osteoporosis was defined by an average vBMD at L1 and L2 < 80 mg/cm^3^. The European spine phantom (ESP 145) was scanned 10 times on each QCT system for quality control. The quality assurance (QA) results showed the ESP vBMD measured at each center differed by less than 5 mg/cm^3^ on average. Therefore, the original vBMD was used for further analysis. Based on 10 repeated scans of the ESP at each participating center the median coefficient of variation (%CV) for the L1–L3 ESP vBMD was 0.48% (range, 0.31% to 1.20%) (11). All data were transferred to the Data Management Center (Beijing Jishuitan hospital) for data cleaning and analysis.

DXA measurements of aBMD and lumbar spine projected area were conducted using GE Lunar DXA (GE Lunar Prodigy and DPX Bravo DXA scanners, GE Healthcare, WI, USA) systems, GE Lunar Encore software and GE Lunar positioning devices to enable consistency and accuracy of patient positioning. The lumbar spine (L1–L4) scan was performed at the Wannan Centre and Guiyang Centre. DXA and LDCT were performed on the same day. Osteoporosis was defined as a T-score < -2.5. All data were transferred to the Data Management Centre (Beijing Jishuitan Hospital) for data cleaning and analysis.

### 2.5 Statistical Analysis

Continuous data were described by the mean and standard deviation (SD), and percentages were calculated for categorical variables. Differences between DM and controls groups were analyzed using student-t tests or Mann-Whitney U tests for continuous variables, and the Chi-square test for categorical variables. General linear models were fitted using the method of least squares to evaluate associations of glucose and vBMD. Both sex-specific continuous variables of glucose and vBMD were evaluated in unadjusted and adjusted general linear models, adjusted by age. To control for the potentially confounding factor of age, propensity score matching (PSM) was applied to match subjects for diabetic patients. The propensity score was calculated with logistic regression and matched using the method of nearest neighbor matching with a caliper of 0.1. The balance test of propensity score matching was carried out by using standard difference. Wilcoxon matched pairs signed rank test was used for the comparison after PSM. Because the age distribution of the study population differed from that of the Chinese population as a whole, the sex-specific prevalence of osteoporosis was standardized using the China Biobank study prevalence for each 2-year age group and the most recent Chinese population data (2010 China Census Data) (11). All statistical analyses were performed using the IBM SPSS Statistic 24 and R 3.64 software. A p-value < 0.05 was taken to be statistically significant.

## 3 Results

### 3.1 Baseline Parameters

#### 3.1.1 QCT Full Cohort

Baseline characteristics of the subjects are presented in **Table 1**. Of the 4420 women, 291 fitted the diagnostic criteria of diabetes (49 by FPG >7.0 mmol/L and 242 by health check records). Women with diabetes were significantly older (63 versus 51 years) and had a higher BMI (24.6 vs 23.1) than the non-diabetes women. Of the 5889 men, 606 fitted the diagnostic criteria of diabetes (163 by FPG >7.0 mmol/L and 443 by health check records). The men with diabetes were significantly older (59 versus 52 years) and had a slightly but significantly higher BMI (25.0 vs 24.5) than the non-diabetes men. Women had a mean vBMD of 135.8 mg/cm^3^ and 12.1% of the women met the definition of osteoporosis (OP), men had a mean vBMD of 130.7 mg/cm^3^ and 6.5% met the definition of osteoporosis. The prevalence of OP was significantly higher in women with diabetes (37.5% vs 10.4%), but following age-standardization using the 2010 China Census Data (11), the estimated prevalence of osteoporosis was similar, the adjusted OP rates for DM women being 12.8% and non-DM women 12.1%.

**Table 1:** Characteristics of participants with and without diabetes mellitus (DM) in the QCT full cohort.

	Women				Men			
	Total	Non-DM	DM	P	Total	Non-DM	DM	P
**N**	4420	4129	291		5889	5283	606	
**Age**	51.5 ± 11.2	50.6 ± 10.6	63.8 ± 11.2	<0.001	52.9 ± 11.8	52.1 ± 11.6	59.4 ± 11.7	<0.001
**Height**	157.6 ± 5.5	157.7 ± 5.5	155.8 ± 5.6	<0.001	168.3 ± 5.7	168.4 ± 5.6	167.1 ± 5.8	<0.001
**Weight**	57.6 ± 7.9	57.4 ± 7.8	59.8 ± 9.2	<0.001	69.7 ± 9.4	69.6 ± 9.4	69.8 ± 9.4	0.731
**BMI**	23.2 ± 3.0	23.1 ± 2.9	24.6 ± 3.2	<0.001	24.6 ± 2.9	24.5 ± 2.9	25.0 ± 3.0	<0.001
**FPG**	5.1 ± 1.0	4.9 ± 0.5	7.6 ± 2.5	<0.001	5.3 ± 1.3	5.0 ± 0.6	7.9 ± 2.4	<0.001
**vBMD**	135.8 ± 44.2	138.4 ± 43.4	99.1 ± 38.4	<0.001^a^;	130.7 ± 34.0	131.7 ± 35.2	121.7 ± 30.8	<0.001^a^;
				0.817^b^				0.288^b^
**N of Osteopenia(%)**	1059(24.0)	959(23.2)	100(34.4)	<0.001^a^	1850(31.4)	1593(30.2)	257(42.4)	<0.001^a^
				0.484^c^				0.105^c^
**N of OP(%)**	537(12.1)	428(10.4)	109(37.5)	<0.001^a^; 0.287^d^	383(6.5)	334(6.3)	49(8.1)	0.095^a^; <0.001^d^

a unadjusted; ^b^adjusted for age; ^c^adjusted for age using the QCT population age’ (adjusted Osteopenia rates for Non-DM women 24.0%, DM women 23.4%, Non-DM men 31.1% and DM men 32.5%, respectively); ^d^adjusted for age using the QCT population age, (adjusted OP rates for Non-DM women 12.1%, DM women 12.8%, Non-DM men 6.9% and DM men 4.5% respectively).

For the total population, among the men 1.5% were underweight (BMI<18.5), 54.6% were normal (BMI 18.5-24.9), 40.3% were overweight (BMI 25-29.9) and 3.6% were obese (BMI 30-39). Among the women, the percentages were 2.9%, 72.4%, 22.3% and 2.4%, respectively.

N, number; BMI, body mass index; FPG, fasting plasma glucose; vBMD, volumetric bone mineral density; OP, osteoporosis.

#### 3.1.2 DXA Subcohort

Baseline characteristics of the subjects are presented in **Table 2**. Of the 444 women, 32 fitted the diagnostic criteria of diabetes. Women with diabetes were significantly older (61 versus 52 years) and had a higher BMI (25.6 vs 23.4) and abdominal total fat area (304 versus 242 cm^2^) than the non-diabetes women. Of the 593 men, 80 fitted the diagnostic criteria of diabetes. The men with diabetes were significantly older (55 versus 50 years) and, although the BMI was similar, the total fat area of the abdomen was significantly higher (295 vs 264 cm^2^) than in the non-diabetes men. Women had a mean aBMD of 1.00 g/cm^2^ and 14.2% of the women met the definition of osteoporosis (OP), men had a mean aBMD of 1.06 g/cm^2^ and 4.9% met the definition of osteoporosis. The prevalence of OP was significantly higher in women with diabetes (40.6%), but following age-standardization using the 2010 China Census Data (11), the estimated prevalence of osteoporosis was similar, the adjusted OP rates for DM women being 10.6% and non-DM women 11.1%.

**Table 2:** Comparisons of participants with DXA-derived aBMD and QCT-derived vBMD between DM and Non-DM in DXA subcohort.

	Women							Men					
	Total	Non-DM	DM	P	p1	p2	Total	Non-DM	DM	P	p1	p2
**N**	444	412	32				593	513	80			
**Age(years)**	52.7 ± 10.1	52.0 ± 9.9	61.2 ± 9.7	<0.001			50.5 ± 9.9	49.9 ± 9.6	54.7 ± 10.3	<0.001		
**Height**	156.0 ± 5.6	156.3 ± 5.5	152.6 ± 5.6	<0.001			168.5 ± 5.7	168.7 ± 5.8	167.3 ± 6.2	0.043		
**Weight**	57.3 ± 8.2	57.1 ± 8.2	59.6 ± 8.6	0.100			70.6 ± 9.5	70.5 ± 9.4	71.1 ± 10.2	0.616		
**BMI**	23.6 ± 3.2	23.4 ± 3.2	25.6 ± 2.9	<0.001			24.8 ± 3.0	24.8 ± 3.0	25.4 ± 3.1	0.094		
**FPG**	5.05 ± 0.84	4.90 ± 0.51	7.04 ± 1.51	<0.001			5.42 ± 1.67	4.97 ± 0.58	8.30 ± 2.94	<0.001		
**TFA(cm^2^)**	246 ± 96	242 ± 95	304 ± 93	<0.001			268 ± 100	264 ± 99	295 ± 104	0.01		
**vBMD(L1-2)**	128 ± 43	131 ± 42	94 ± 35	<0.001	0.139	0.254	130 ± 31	131 ± 31	125 ± 27	0.124	0.471	0.367
**aBMD(L1-2)**	1.00 ± 0.17	1.01 ± 0.16	0.89 ± 0.16	<0.001	0.292	0.195	1.06 ± 0.14	1.06 ± 0.13	1.09 ± 0.15	0.034	0.022	0.064
**N of OP(%)**	63(14.2)	50(12.1)	13(40.6)	<0.001^a^; 0.459 ^b^			29(4.9)	25(4.9)	4(5.0)	0.961^a^; <0.001^b^		

a unadjusted; ^b^adjusted for age using the QCT population age, (adjusted OP rates for Non-DM women 11.1%, DM women 10.6%, Non-DM men 5.9%, DM men 2.2%, respectively).

p1: adjusted for age.

p2: adjusted for age and TFA.

TFA, total fat area of the abdomen at L2 level; aBMD, areal bone mineral density.

### 3.2 Association of BMD With FPG

#### 3.2.1 QCT Full Cohort

**Figure 1:**
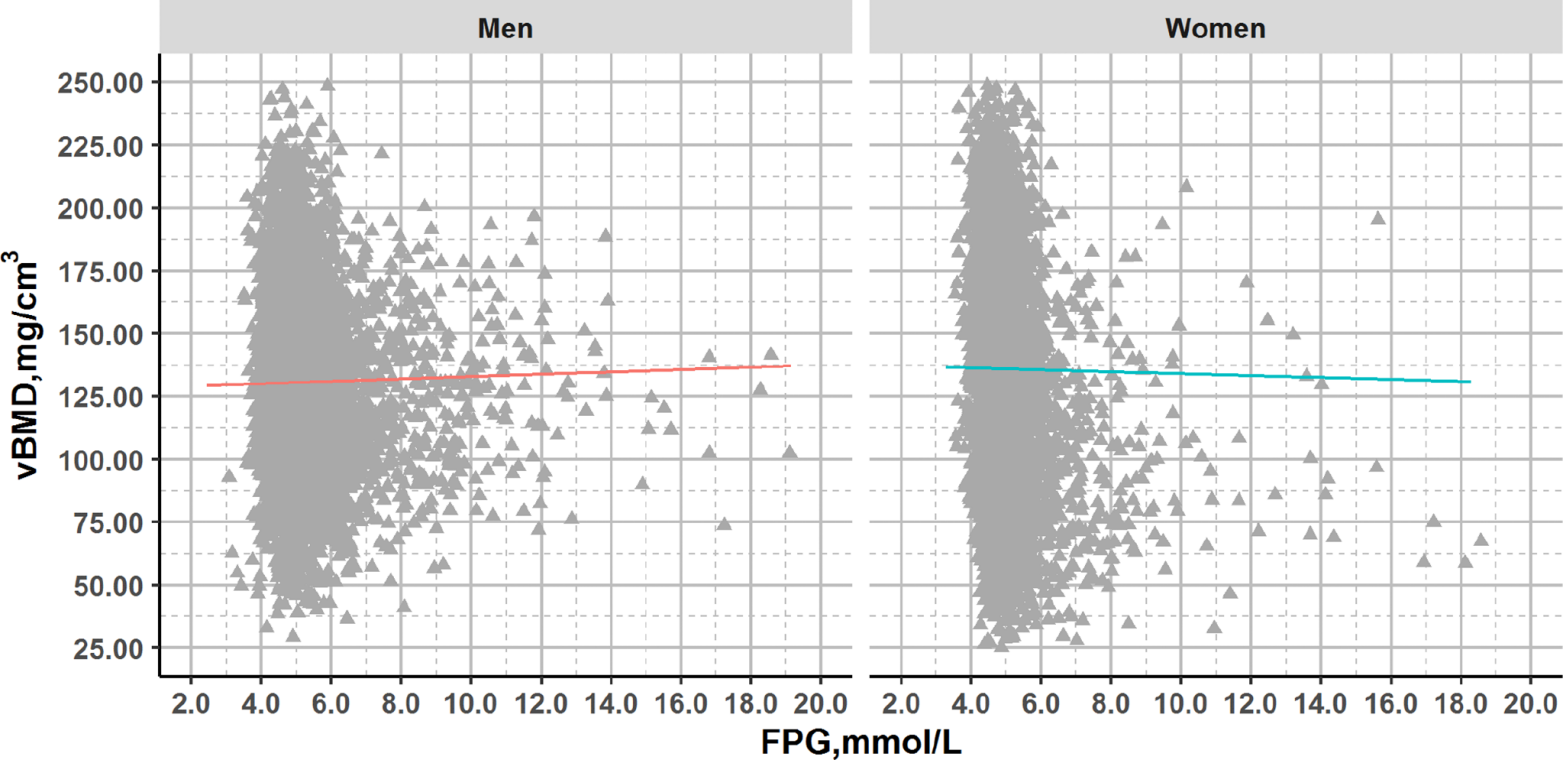
Plots of FPG and vBMD in QCT full cohort with glucose concentrations across the range from normal to diabetes. Association lines (adjusted for age): Men: y=0.431x+128.501, R^2^ = 0.000, p>0.05; Women: y=-0.125x+136.574, R^2^ = 0.000, p>0.05.

There was no significant association between vBMD and FPG after adjustment for age (men: p=0.149; women: p=0.774) (**Figure 1** and **Figures S1**, **S2**).

#### 3.2.2 DXA Subcohort

**Figure 2:**
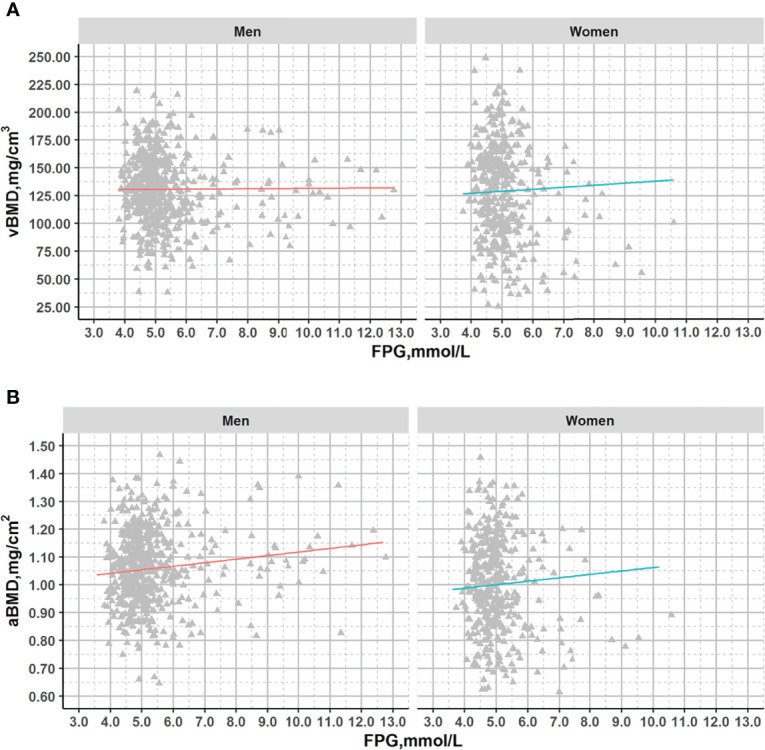
Plots of vBMD and aBMD with fasting glucose across the range from normal to diabetes in DXA subcohort. Association lines (adjusted for age): **(A)**. Men: y=-0.190x+131.714, R^2^ = 1.49*10^-4^, p=0.775; Women: y=1.964x+119.133,R^2^ = 0.003,p=0.304; **(B)**. Men: y=0.009x+1.013, R^2^ = 0.011,p=0.016; Women: y=0.012x+0.944, R^2^ = 0.004,p=0.203;.

After adjustment for age, a significant association with FPG was observed for aBMD in men (p=0.011) but not in women (p=0.203) or for vBMD (men: p=0.775; women: p=0.403) (**Figure 2**).

### 3.3 Comparison of BMD Between Diabetes Patients and Healthy Subjects

#### 3.3.1 QCT Full Cohort

**Table 1** shows that subjects with diabetes have lower vBMD compared to healthy subjects in both men and women. However, after adjusting for age, no significant difference was observed. To investigate this further, we used propensity score matching. After PSM using age, 277 women with type 2 diabetes mellitus were matched with 277 healthy controls and 592 type 2 diabetes mellitus men with 592 healthy controls (**Table 3**). **Table 3** also shows that, as expected, type 2 diabetes mellitus patients have higher BMI and FPG although the differences are small. In the 277 women with FPG concentrations in the diabetic range (7.6 ± 2.5 mmol/L) compared to women with FPG concentrations in the normal range (5.0 ± 0.5 mmol/L) (**Table 3**), vBMD was not significantly different (99.8 vs 99.6 mg/cm^3^, p=0.678). In 592 men matched for age with fasting plasma glucose levels in the normal (5.1 ± 0.6 mmol/L) versus in the diabetic (7.9 ± 2.3 mmol/L) range, vBMD was also not significantly different (119.4 vs 121.5 mg/cm^3^, p=0.135).

**Table 3:** Characteristics of matched participants with and without DM using propensity score in the QCT full cohort.

	Women			Men		
	Non-DM	DM	P	Non-DM	DM	P
**N**	277	277		592	592	
**Age**	63.6 ± 10.9	63.6 ± 10.9	1.000	59.3 ± 11.6	59.3 ± 11.6	1.000
**BMI**	23.7 ± 3.0	24.6 ± 3.2	0.001	24.1 ± 2.9	25.0 ± 3.0	<0.001
**FPG**	5.0 ± 0.5	7.6 ± 2.5	<0.001	5.1 ± 0.6	7.9 ± 2.3	<0.001
**vBMD**	99.8 ± 42.2	99.6 ± 38.7	0.678	119.4 ± 33.7	121.5 ± 30.8	0.135
**N of OP (%)**	96(34.7)	104(37.5)	0.479	62(10.5)	48(8.1)	0.161

Propensity score matching by age.

#### 3.3.2 DXA Subcohort

**Table 2** demonstrates comparisons of participants with DXA-derived aBMD and QCT-derived vBMD between DM and non-DM. Before adjusting for age, interestingly both vBMD and aBMD were significantly lower in women with diabetes compared to non-diabetic women whereas, as expected according to our hypothesis, vBMD was lower but aBMD was higher in men with diabetes compared to men without diabetes. After adjusting for age, only the aBMD remained significantly higher in men with diabetes. But finally, after adjusting for total fat area, also this difference disappeared.

After PSM using age, 29 women with type 2 diabetes mellitus were matched with 29 healthy controls and 79 type 2 diabetes mellitus men with 79 healthy controls (**Table 4**). **Table 4** also shows that, as expected, type 2 diabetes mellitus patients have higher BMI and FPG although the differences are small. In the 277 women with FPG concentrations in the diabetic range (7.6 ± 2.5 mmol/L) compared to women with FPG concentrations in the normal range (5.0 ± 0.5 mmol/L) (**Table 3**), vBMD as well as aBMD were not significantly different (vBMD 100.5 vs 98.4 mg/cm^3^, p=0.738; aBMD 0.91 vs. 0.91, p=0.430). In 79 men matched for age with fasting plasma glucose levels in the normal (5.0 ± 0.6 mmol/L) versus in the diabetic (8.2 ± 2.9 mmol/L) range, vBMD was not significantly different (vBMD: 119.4 vs 121.5 mg/cm^3)^, however the aBMD showed a trend towards higher aBMD in in men (aBMD 1.05 vs. 1.09, p=0.074). In addition, we used PSM with age and BMI to clarify the role of fat tissue and in men, the difference indeed disappeared (aBMD 1.08 vs. 1.09, p=0.903).

**Table 4:** Characteristics of matched participants with and without DM using propensity score in the DXA subcohort.

	Matched population^1^						Matched population^2^					
	Women			Men			Women			Men		
	Non-DM	DM	P	Non-DM	DM	P	Non-DM	DM	P	Non-DM	DM	P
**N**	29	29		79	79		30	30		79	79	
**age**	59.8 ± 8.7	59.8 ± 8.7	1.000	54.3 ± 9.7	54.3 ± 9.7	0.705	61.0 ± 8.6	60.8 ± 9.7	0.665	55.3 ± 10.6	54.5 ± 10.2	0.359
**BMI**	23.26 ± 2.61	25.73 ± 2.85	0.003	24.42 ± 2.98	25.40 ± 3.09	0.048	24.29 ± 2.83	25.35 ± 2.87	0.075	24.76 ± 2.62	25.33 ± 3.12	0.173
**FPG**	5.10 ± 0.68	6.85 ± 1.60	<0.001	4.97 ± 0.63	8.19 ± 2.94	<0.001	5.08 ± 0.56	6.66 ± 1.52	<0.001	5.08 ± 0.64	8.15 ± 2.95	<0.001
**TFA**	260.67 ± 82.51	314.76 ± 84.49	0.048	243.59 ± 98.27	296.13 ± 104.63	0.005	292.22 ± 83.79	293.75 ± 86.00	0.781	280.01 ± 97.88	293.03 ± 103.14	0.395
**vBMD**	100.53 ± 43.38	98.39 ± 34.13	0.738	127.35 ± 29.75	125.52 ± 28.78	0.642	104.88 ± 34.31	94.09 ± 36.37	0.141	127.46 ± 34.31	125.71 ± 28.69	0.528
**aBMD**	0.91 ± 0.20	0.91 ± 0.16	0.430	1.05 ± 0.14	1.09 ± 0.16	0.074	0.92 ± 0.15	0.89 ± 0.17	0.658	1.08 ± 0.15	1.09 ± 0.16	0.903

^1^PSM by age; ^2^PSM by age and TFA.

## 4 Discussion

As a main result we did not find any association of vBMD with fasting plasma glucose across the healthy to diabetic range in this large cohort of >10.000 subjects. Also, when comparing subjects with and without diabetes, vBMD was similar after adjustment for age in men and women. Considering that diabetes nowadays is a prevalent condition worldwide with ever increasing numbers, it is important to be able to adequately predict fracture risk, initiate treatment and prevent fractures in these patients (12). This study confirms that vBMD measured with QCT is not affected by diabetes or fasting plasma glucose concentration i.e. does not overestimate BMD and therefore could be used as a reliable estimate of BMD to assess fracture risk in diabetes.

Although the diagnosis of diabetes is defined by a fasting plasma glucose above a threshold of 7.0 mmol/L, there is a continuum of fasting plasma glucose concentrations from normal to impaired fasting glucose (IFG) to diabetic where the risk of diabetes complications is progressively increasing with increasing fasting plasma glucose concentrations (13, 14). In addition, many patients are unaware of their diabetes for years and are often diagnosed by screening or based on the manifestation of complications. Indeed, a recent study showed that in 170.000 Chinese subjects with a mean age of 44 years, the rate of diabetes based on HbA1c measurements was 10.9% of which only 4% was previously diagnosed and 38% fitted the diagnosis of prediabetes (15).

Most studies assessing BMD and diabetes/glucose measured areal BMD with DXA instead of QCT and reported a higher BMD in diabetes subjects (1, 3, 5, 6, 16–18). In our QCT full cohort, a subgroup of subjects also underwent DXA scanning in addition to QCT. QCT results in this DXA subcohort were comparable to those in the full cohort. Interestingly, in these subjects, we found a higher aBMD only in men but the number of women with diabetes was small (N=29) precluding any conclusions from these data. In men (N=79), we confirmed the higher aBMD, also after adjustment for age. Since QCT scans were available of these patients, we could measure total fat area of the abdomen (TFA) with this state-of-the-art technique (19) and we showed that after adjustment for TFA the higher aBMD in men was indeed no longer significantly different between diabetic and non-diabetic subjects. This result, although obtained in a small group of subjects, indeed supports the common notion of the overestimation of aBMD due to overlying soft tissue. A very recent study showed that diabetes increased aBMD by increasing obesity-related indexes (20). Several other studies also indicated that aBMD was associated with BMI and that differences in aBMD between diabetic and nondiabetic subjects disappeared after adjustment for BMI (21–23).

In the large Diabetes Heart Study BMD was measured by DXA and QCT (22). There was a very weak correlation of vBMD measured at the lumbar spine with BMI but in the total cohort there was no difference in age adjusted vBMD of the lumbar spine between diabetic (T2DM, n=808) and non diabetic (n=106) subjects. In contrast the age adjusted aBMD difference of the lumbar spine and total hip was significant and in agreement with our results and the studies cited above disappeared after adjustment for BMI.

Our results are similar to another recent QCT-based study in a Chinese population of 4000 subjects of which 600 had diabetes, showing that, without adjustment for age, vBMD was lower in impaired fasting glucose and diabetic patients. Unfortunately, age adjusted data were not presented in that study although there was a significant age difference between the groups (normal 47 years, IFG 53, diabetes 55 years) (24) and many studies including the current one have shown significant vBMD decreases with age (**Figure S1**).

In contrast to the existing literature and to our results in men, we did not find a positive association between areal BMD and glucose in women or a higher areal BMD in women with diabetes, although the number of women in the latter analysis was small (N=29) and needs to be interpreted with caution. This is a limitation of our study. Several factors could explain this sex difference and/or incongruity within the literature; I] sex and menopausal status; BMD accrual, peak bone mass and bone loss are different between men and women and menopause has a profound effect on bone remodeling, therefore analyses should be stratified for age and menopausal status. II] ethnicity and BMI; although the prevalence of diabetes is comparable in Western and Chinese societies, the BMI at which patients develop diabetes is very different and perhaps more importantly BMI does not capture differences in body composition (15, 25). Only adjustment for TFA but not for BMI eliminated aBMD differences between men with and without diabetes. It is important to note that characteristics of the DM population in our study were similar to DM patients across China, which were characterized in a recent study (15). III] age is an important determinant of BMD and diabetes becomes more likely with aging. Therefore adjusting analyses for age is of paramount importance IV] diabetes duration, treatment and glycemic control; many complications of diabetes tend to become more frequent with longer duration of diabetes and poorer glycemic control (7, 13, 26). In addition, diabetes treatment such as insulin or thiazolidinediones can also impact on bone mass. It is another limitation of our study that data on the duration or diabetic treatment were missing in our cohort, but we do conclude from our data that most patients were well controlled considering their mean fasting plasma glucose of 7.6 mmol/L. V] location of BMD measurement; BMD is commonly measured at the spine or hip, however these sites are not interchangeable and can be differentially affected in certain disease states depending on the effect on trabecular (spine) or cortical (hip) bone of the underlying disease (4, 27). As another limitation, in this study, we had only spine BMD data available. Given the fact that vertebral fracture risk in type 2 diabetes mellitus diabetes is not or only marginally increased, the hip may be the preferred anatomical site to assess. VI] type of diabetes; type 1 diabetes patients have a lower areal and volumetric BMD and a much higher fracture risk than type 2. It is also a limitation that we did not have data available on diabetes type of our patients, type 2 diabetes is much more common than type 2 (95% versus 5% of all diabetes patients), therefore we assumed that the vast majority of our patients would be type 2.

However, strengths of our study include the large number of subjects included in this cohort (>10.000) and the state-of-the-art measurement technique of vBMD and TFA with QCT.

In conclusion, we did not find any effect of fasting plasma glucose or diabetes on the volumetric BMD measured with QCT after adjustment for age. Therefore, without additional adjustments for body composition, vBMD measured with QCT might be a more reliable measurement to assess osteoporosis and fracture risk than aBMD measured with DXA in diabetic patients.

## Data Availability Statement

The datasets presented in this article are not readily available because the data for The China Biobank are not presently available to be shared. These data might be available at a future stage. Requests to access the datasets should be directed to xiao65@263.net.

## Ethics Statement

The studies involving human participants were reviewed and approved by Beijing Jishuitan Hospital. The patients/participants provided their written informed consent to participate in this study.

## Author Contributions

LW, XZ, LR, AV, and XC designed the study. KZ led the analysis with input from LW, HS, XZ, LR, LX, AV, KE, and YY. YL, LX, and QS did the literature search. YY, QS, and YL collected the data. YY and QS did the measurements. All authors contributed to data interpretation. LW and KZ wrote the manuscript and all authors reviewed the manuscript.

## Funding

This work is supported in part by Beijing Hospitals Authority Clinical Medicine Development of Special Funding Support (code: ZYLX202107), Beijing Hospitals Authority Youth Programme (code: QMS20200402), and the National Natural Science Foundation of China (Grant Nos. 81901718, 81771831).

## Conflict of Interest

KE is a part-time employee of BioClinica, Inc.

The remaining authors declare that the research was conducted in the absence of any commercial or financial relationships that could be construed as a potential conflict of interest.

## Publisher’s Note

All claims expressed in this article are solely those of the authors and do not necessarily represent those of their affiliated organizations, or those of the publisher, the editors and the reviewers. Any product that may be evaluated in this article, or claim that may be made by its manufacturer, is not guaranteed or endorsed by the publisher.
