# Supplementary material for: Hyperglycemia Is Not Associated With Higher Volumetric BMD in a Chinese Health Check-up Cohort
Source: Front Endocrinol (Lausanne). 2022 Jan 3;12:794066. doi: 10.3389/fendo.2021.794066 (PMC8763321; doi:10.3389/fendo.2021.794066)
Supplement: Supplementary file 1 [file DataSheet_1.docx]

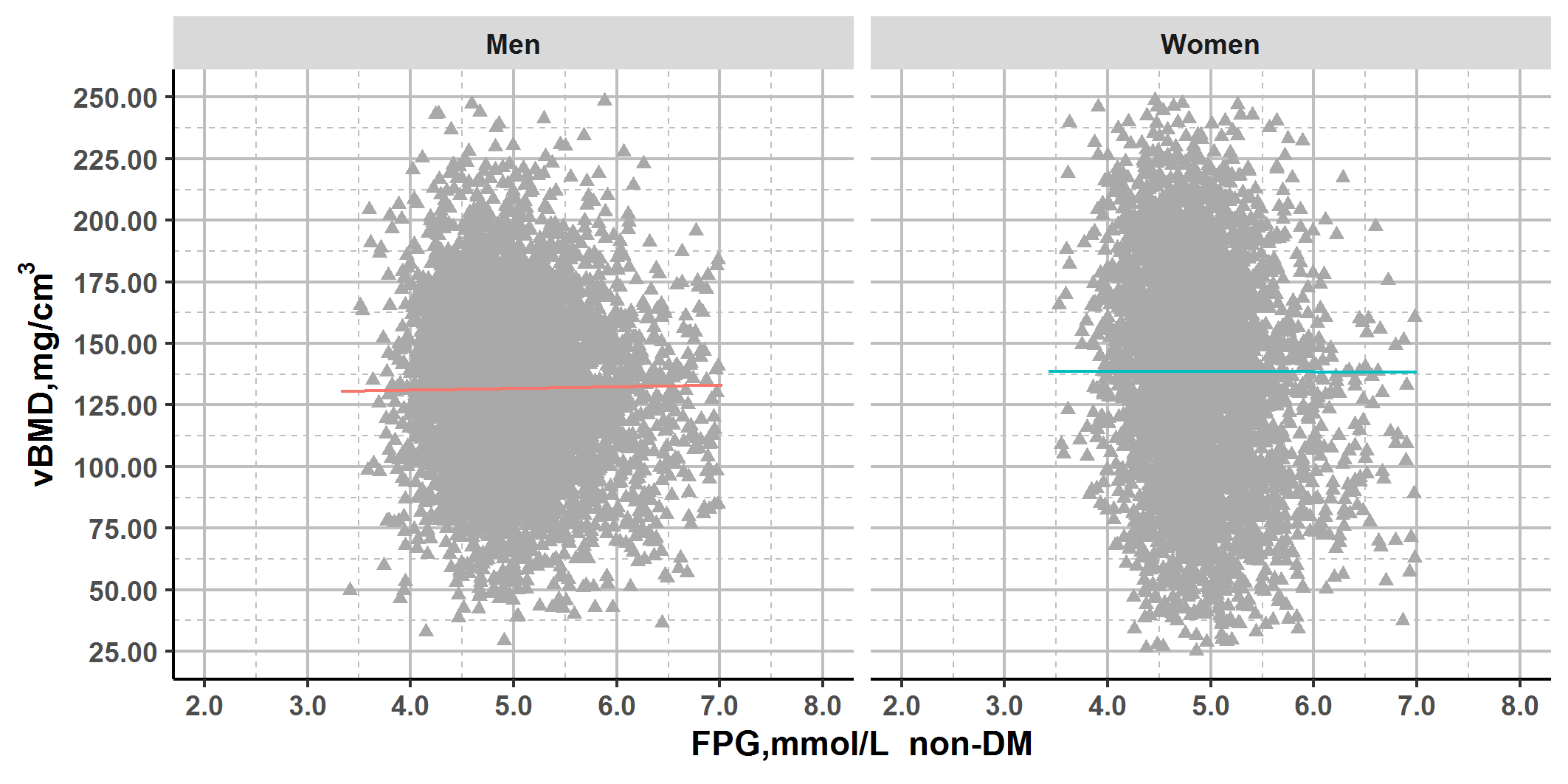


**Figure S1** Relation of vBMD and FPG in QCT cohort without diabetes after adjustment for age

Men: y=0.60x+129, R^2^=0.000; Women: y=-0.028x+100, R^2^=0.000.


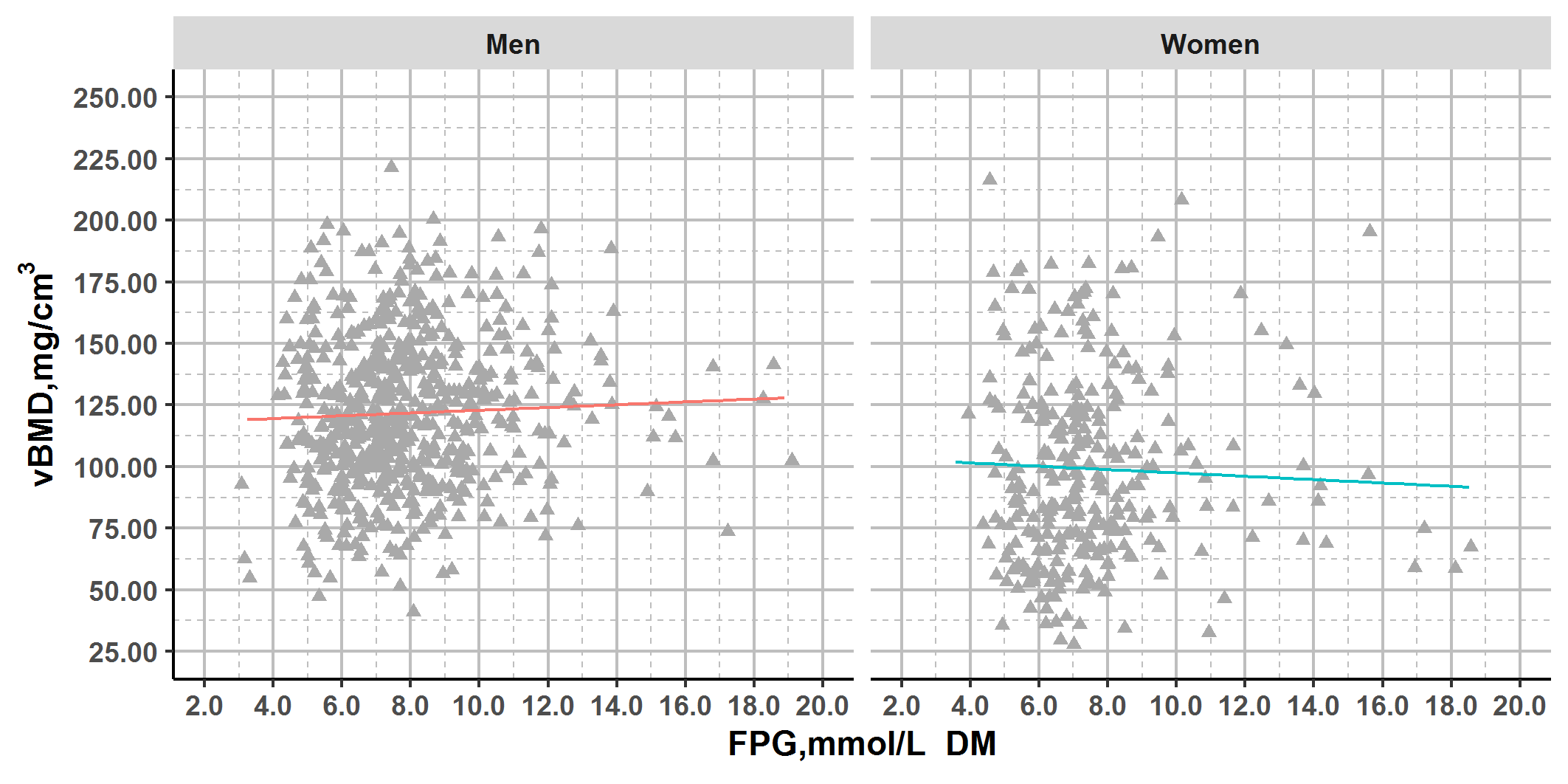


**Figure S2** Relation of vBMD and FPG in QCT cohort with diabetes after adjustment for age

Men: y=0.59x+117, R^2^=0.003; Women: y=-0.087x+100, R^2^=0.000.


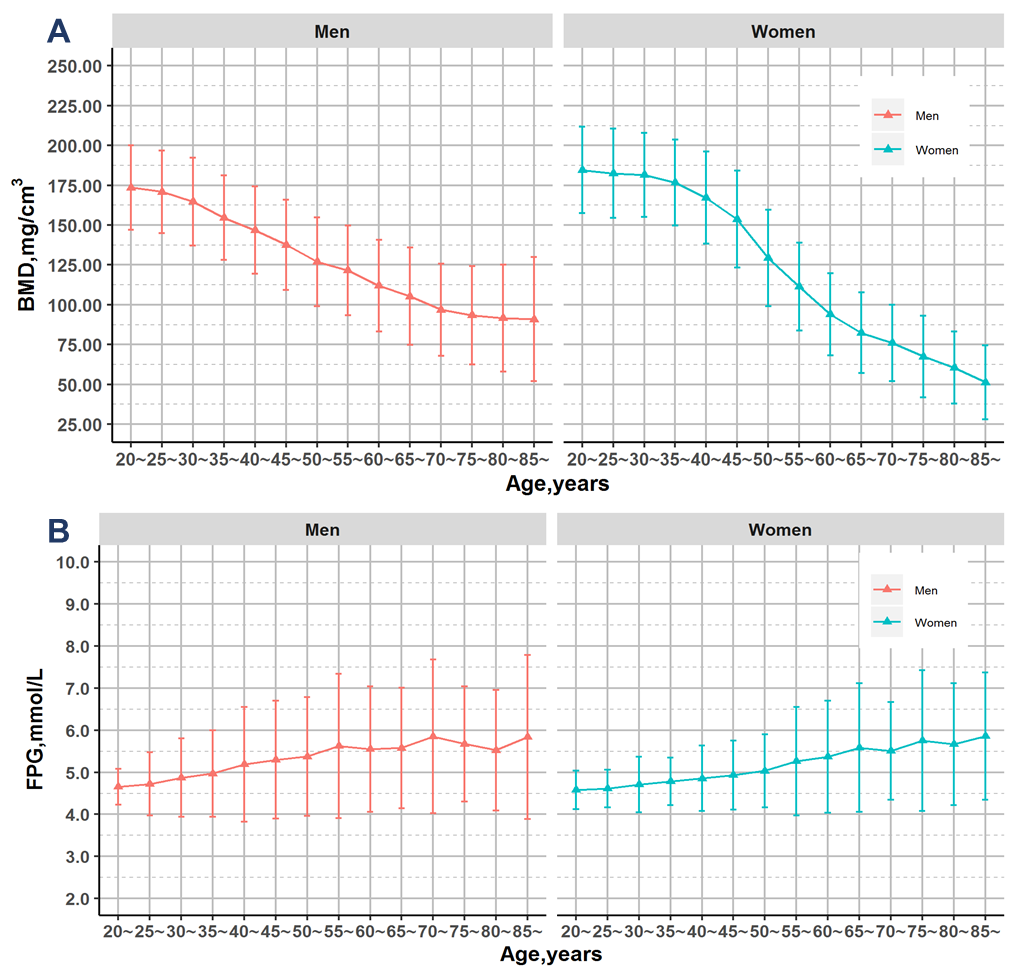


**Figure S3.** The mean and SD of BMD and FPG variations with age for 10309 participants.


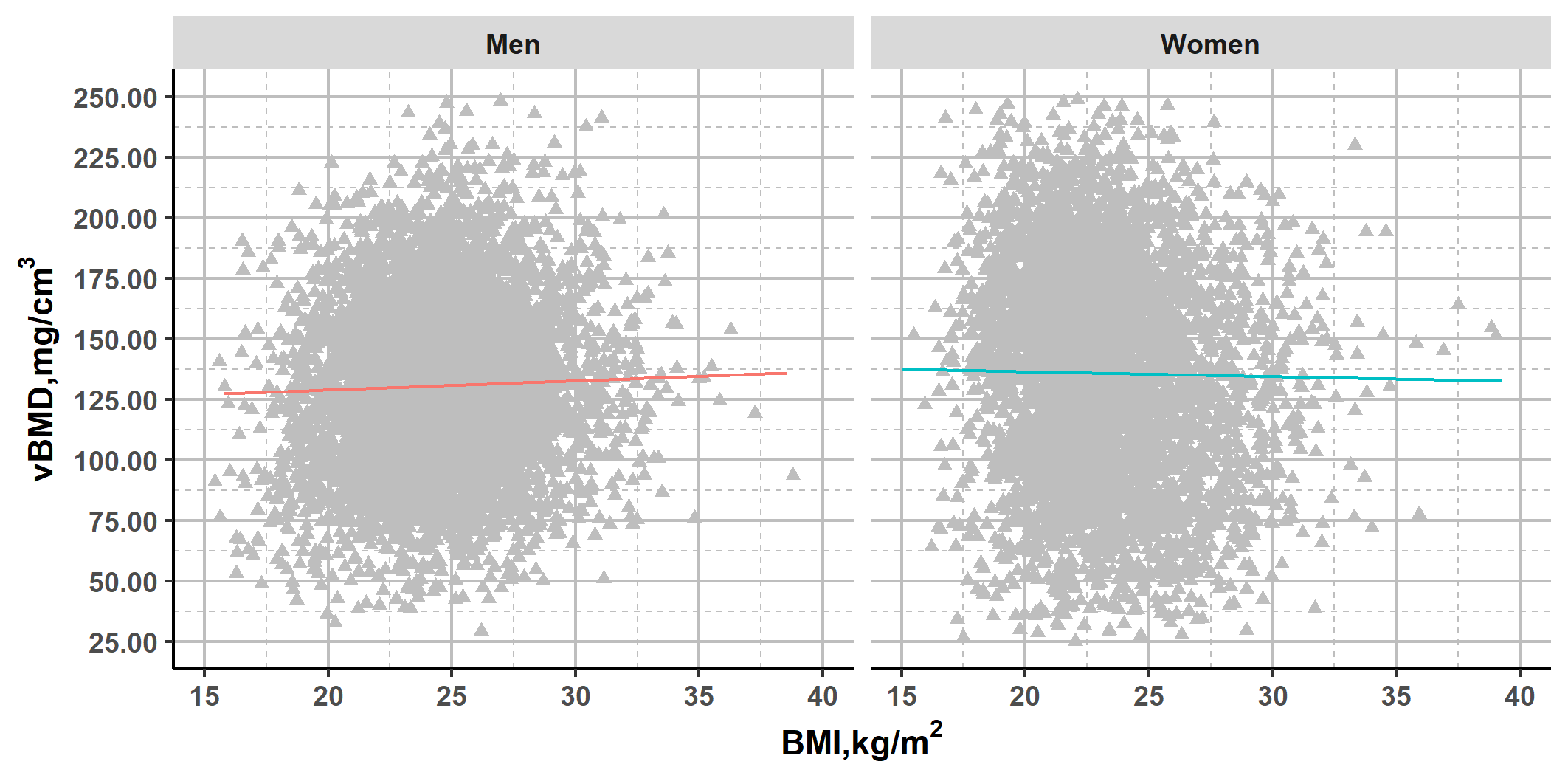


**Figure S4** Plots of BMI and vBMD in QCT full cohort with glucose concentrations across the range from normal to diabetes.

Notes: Association lines (adjusted for age):

Men: y=0.39x+121, R^2^=0.002, p=0.003; Women: y=-0.20x+140, R^2^=0.000, p=0.187
